# Plastid Envelope-Localized Proteins Exhibit a Stochastic Spatiotemporal Relationship to Stromules

**DOI:** 10.3389/fpls.2018.00754

**Published:** 2018-06-04

**Authors:** Kathleen Delfosse, Michael R. Wozny, Kiah A. Barton, Neeta Mathur, Nigel Griffiths, Jaideep Mathur

**Affiliations:** Laboratory of Plant Development and Interactions, Department of Molecular and Cellular Biology, University of Guelph, Guelph, ON, Canada

**Keywords:** plastids, stromules, fluorescent proteins, transporters, chloroplast proteins

## Abstract

Plastids in the viridiplantae sporadically form thin tubules called stromules that increase the interactive surface between the plastid and the surrounding cytoplasm. Several recent publications that report observations of certain proteins localizing to the extensions have then used the observations to suggest stromule-specific functions. The mechanisms by which specific localizations on these transient and sporadically formed extensions might occur remain unclear. Previous studies have yet to address the spatiotemporal relationship between a particular protein localization pattern and its distribution on an extended stromules and/or the plastid body. Here, we have used discrete protein patches found in several transgenic plants as fiducial markers to investigate this relationship. While we consider the inner plastid envelope-membrane localized protein patches of the GLUCOSE 6-PHOSPHATE/PHOSPHATE TRANSLOCATOR1 and the TRIOSE-PHOSPHATE/ PHOSPHATE TRANSLOCATOR 1 as artifacts of fluorescent fusion protein over-expression, stromule formation is not compromised in the respective stable transgenic lines that maintain normal growth and development. Our analysis of chloroplasts in the transgenic lines in the Arabidopsis Columbia background, and in the *arc6* mutant, under stromule-inducing conditions shows that the possibility of finding a particular protein-enriched domain on an extended stromule or on a region of the main plastid body is stochastic. Our observations provide insights on the behavior of chloroplasts, the relationship between stromules and the plastid-body and strongly challenge claims of stromule-specific functions based solely upon protein localization to plastid extensions.

**One sentence summary:**

Observations of the spatiotemporal relationship between plastid envelope localized fluorescent protein fusions of two sugar-phosphate transporters and stromules suggest a stochastic rather than specific localization pattern that questions the idea of independent functions for stromules.

## Introduction

Plastids in green plants and algae are bound by a double-membrane envelope and sporadically extend and retract thin tubules (Wildman et al., 1962; Wise, 2007; Pyke, 2009). The tubules were first highlighted using a stroma-targeted Green Fluorescent Protein (GFP; Köhler et al., 1997), and consequently were called stroma-filled tubules or stromules (Köhler and Hanson, 2000). While only a small subset of the total plastid population exhibits stromules during normal growth and development, a range of conditions are known to increase the incidence of stromule formation in a cell (Natesan et al., 2005; Hanson and Sattarzadeh, 2011; Schattat et al., 2011a,b, 2015; Krenz et al., 2012; Hanson and Hines, 2018). Since their first visualization the function of stromules has been the subject of several conjectures and debates (Köhler and Hanson, 2000; Kwok and Hanson, 2004a; Newell et al., 2012; Schattat et al., 2012a,b, 2015; Hanson and Hines, 2018). Observing stromules extending from the main plastid body strongly suggests that their presence increases the interactive surface between the plastid and the neighboring cytoplasm. These observations form the basis for believing that stromules facilitate the bi-directional trafficking of proteins and metabolites.

Indeed, a role for stromules has been suggested in the flux of the mono-terpenoid geraniol (Simkin et al., 2013), the localization of geranylgeranyl diphosphate synthase (Thabet et al., 2012), and in the re-localization of the NUCLEAR RECEPTOR INTERACTING PROTEIN 1 from the nucleus to plastids during pathogen response (NRIP1; Caplan et al., 2008; Krenz et al., 2012). Several reports appear to consider stromules as separate domains of the plastid and place considerable emphasis upon the observation that the particular protein under consideration localizes to stromules which belies the fact that the considered protein is localized to the stroma and is thus evenly distributed throughout the plastid's body and stromule (Wang et al., 2004, 2017; Howes, 2013; Rad and Kohalmi, 2015; Aranda-Sicilia et al., 2016; Bross et al., 2017). Notably, these studies do not provide any explanation as to why they choose to make a distinction between the continuous plastid stroma and the stroma-filled tubule. To the best of our knowledge there is no ultra-structural or biochemical evidence that compartmentalizes the stroma in a manner that would allow a stromule to be considered a sub-structure of the plastid.

More recently, observations from an approach combining proteomics and transient protein localization data in *Physcomitrella patens* have been used to suggest very specific roles for stromules that implicate them in fatty acid biosynthesis, redox homeostasis, and metabolite transport (Mueller and Reski, 2014; Mueller et al., 2014). Mueller et al. (2014) conclude that, “stromules are micro-compartments of plastids that accumulate specific proteins to serve specialized functions.” This statement is quite appealing as it goes along with the basic concept of micro-domains and dynamic compartmentation as being fundamental for the make-up and division of function within the eukaryotic cell (Pielak, 2005; Vesteg et al., 2006). Indeed, the vast majority of plastid proteins are encoded by genes in the nucleus and post-translationally targeted to one of several discrete domains in the organelle, such as the envelope membranes, the stroma, thylakoid membranes, and the thylakoid lumen (Soll and Tien, 1998; Keegstra and Froehlich, 1999; Schleiff and Soll, 2000). Specific proteins also localize to plastid DNA nucleoids (Terasawa and Sato, 2005; Melonek et al., 2012), and to inclusions such as starch grains (Christiansen et al., 2009; Szydlowski et al., 2009), and plastoglobuli (Shumskaya et al., 2012; Gámez-Arjona et al., 2014). Often a strong biochemical basis is provided to support the protein localization data.

Therefore, a point that becomes debatable is whether stromules, transient extensions observed sporadically, should at all be considered a plastid sub-compartment. More important, since plastids normally do not exhibit stromules all the time, it is perplexing as to how a protein might become targeted specifically to a stromule? Could the protein be residing in another plastidial location, and then, under certain conditions become localized to a stromule? These questions require a thorough study of the spatiotemporal aspects of protein localization with reference to the plastid body and the extended stromule. Fiducial markers that can allow such an investigation are singularly lacking. However, several plastidial proteins have been shown to localize as punctae or patches (Lee et al., 2001; Xu et al., 2005; Awai et al., 2006; Haswell and Meyerowitz, 2006; Seo et al., 2009; Tan et al., 2011; Liang et al., 2017; Li et al., 2017; Wang et al., 2017). While many of the reports have relied on transient protein over-expression in heterologous systems for their observations, stable transgenic lines that maintain a rather specific localization pattern for the fusion proteins, such as the poles of chloroplasts (Aranda-Sicilia et al., 2016), or clusters in the stroma (Jiang et al., 2017), have also been reported. We considered the possibility that stable transgenic lines that exhibit discrete fluorescent protein patches on the chloroplast envelope can serve as useful fiducial markers for assessing protein localization to different regions, including the extensions, of a chloroplast.

Based on earlier reports (Xu et al., 2005; Aranda-Sicilia et al., 2016), we considered it very plausible that some proteins, known to be essential for plastid functions, might actually localize as small domains. Further, we speculated that such domains of these important proteins might localize preferentially to the plastid extensions as this could increase their outreach and interactivity with other cytosolic and organelle-resident proteins. We therefore searched for stable transgenic lines that exhibited discrete protein patches and narrowed our search according to the following criteria.

Since a stromule, by definition, constitutes a stroma filled tubule (Köhler and Hanson, 2000) the presence of a stromal-protein in it is to be expected and cannot be used to investigate a stromule-specific localization. Therefore, in choosing candidates for our investigation we excluded stromal proteins. Similarly, proteins known to reside in the plastid outer-envelope membrane were not considered as their over-expression often results in ectopic tubular membrane protrusions that might exhibit a morphological similarity to stromules (Breuers et al., 2012), but might not always be filled by the stroma (Maggio et al., 2007; Oikawa et al., 2008; Machettira et al., 2012; Delfosse et al., 2016). We chose to screen stable transgenic lines of several biochemically well-characterized sugar-phosphate transporters that reside on the inner envelope-membrane and are essential for normal plastid functions (Flügge, 1999; Ferro et al., 2002; Niittylä et al., 2004; Weber and Linka, 2011).

Three transgenic Arabidopsis lines exhibited the desired punctate localization on chloroplasts and were chosen for use as fiducial marker lines for our investigations.

## Results

### Punctate protein dispersal pattern provided fiducial markers on the chloroplast envelope

**Figure 1:**
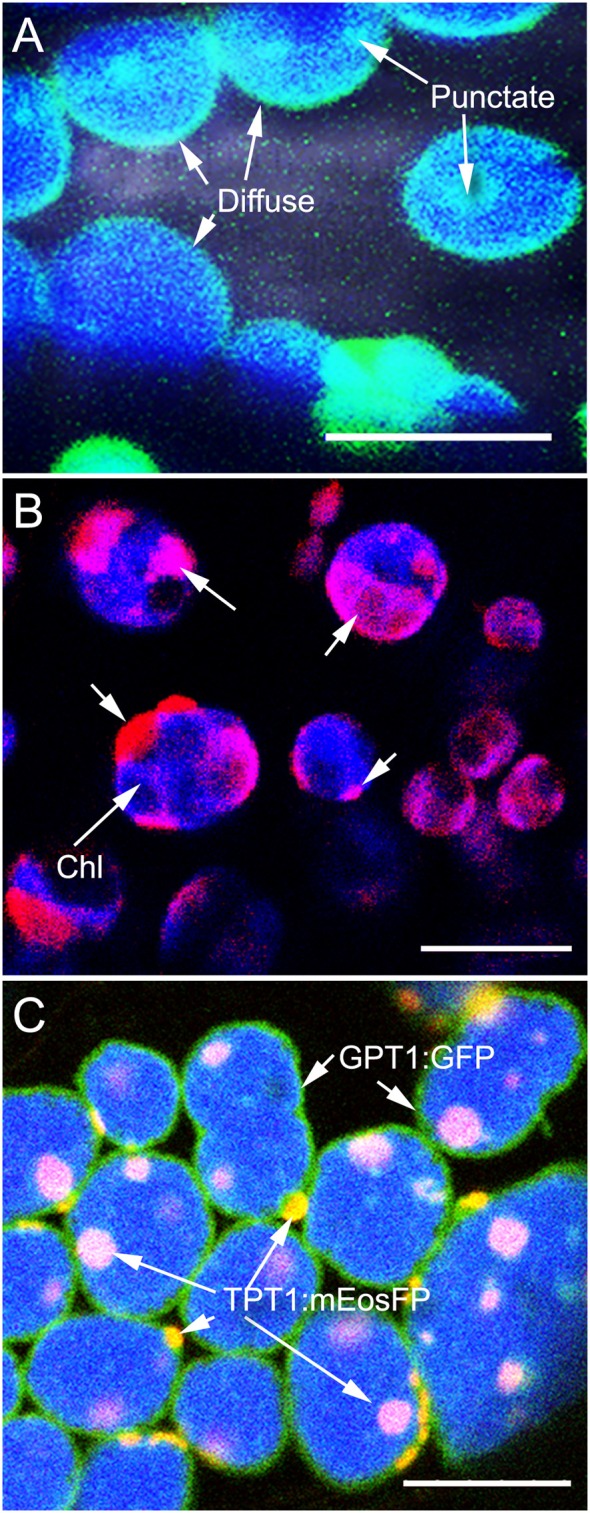
Inner envelope-localized GPT1 and TPT1 fluorescent fusion proteins showing diffuse and punctate dispersal patterns on chloroplasts. **(A)** Mesophyll chloroplasts in an Arabidopsis line expressing GPT1-GFP shows the diffuse highlighting of the entire plastid periphery along with a few, discrete, more fluorescent patches. **(B)** Representative image showing mesophyll chloroplasts with a predominantly patchy localization of GPT1-mEosFP (arrows) upon transient expression in tobacco leaves. **(C)** Image from a double transgenic Arabidopsis line obtained through a genetic cross between homozygous pro35S:GPT1-EGFP (diffuse highlighting) and a pro35S:TPT1-mEosFP (punctate localization). The line expressed both probes with up to 60% of chloroplasts exhibiting the two protein dispersal patterns. Chlorophyll (chl) depicted in blue color. Size bars A, B, C = 10 μm.

Protein dispersal patterns on the plastid inner envelope-membrane can be observed as diffuse when it highlights the entire envelope, or punctate when only a small region of the envelope usually, 0.5–1.5 μm in diameter, becomes highlighted. When the regions highlighted by the fluorescent protein are larger than 1.5 μm we have called them as patches. Both diffuse and punctate protein dispersal patterns were observed in several transient overexpression experiments using different colored fluorescent protein fusions of the plastid inner envelope-membrane localized transporters, TPT1 (TRIOSE-PHOSPHATE / PHOSPHATE TRANSLOCATOR1; Preiss, 1984; Flügge et al., 1989; Flügge and Heldt, 1991), the GPT1 (GLUCOSE 6-PHOSPHATE/PHOSPHATE TRANSLOCATOR1; Fliege et al., 1978; Kammerer et al., 1998, PPT1 (PHOSPHOENOL PYRUVATE / PHOSPHATE translocator1; Fischer et al., 1997); XPT1 (XYLULOSE 5-P/PHOSPHATE TRANSLOCATOR 1; Eicks et al., 2002; Knappe et al., 2003) and the MEX1 (MALTOSE EXCESS 1 transporter; Niittylä et al., 2004). Observations of punctate or patchy localization were considered artifacts of transient protein overexpression. Stable transgenic lines were created in a tpFNR-EGFP background that highlights the plastid stroma in green fluorescence (Marques et al., 2003). Whereas, the majority of chloroplasts in the transporter transgenic lines exhibited a diffuse highlighting of the inner envelope-membrane, in some cases there were also some small fluorescent punctae (Figure 1A). Lines expressing the GPT1-mEosFP also showed this mixed pattern of protein dispersal (Figure 1B) and a single stable transgenic line expressing GPT1-mEosFP displayed a predominantly patchy localization on chloroplasts. A similar line with fusion protein patches was selected from the TPT1-mEosFP transgenics.

In order to assess the stability of the fluorescent patches a cross was created between a homozygous pro35S:GPT1-EGFP, that shows a predominantly diffuse localization and a pro35S:TPT1-mEosFP cross. In the double transgenic line the TPT1-mEosFP fusion localized mostly in the form of discrete patches that, following photo-conversion, were clearly distinguishable from the green fluorescence of the GPT1-EGFP fusion (Figure 1C). Another cross was carried out between the GPT1-mEosFP line and an *arc6* mutant (*accumulation and replication of chloroplasts6*; Pyke et al., 1994) line expressing the tpFNR-EGFP probe. The resultant line also displayed discrete photo-convertible fluorescent protein patches and provided an additional fiducial marker to assess patch localization patterns in the large chloroplasts that are characteristic of the *arc6* mutant (Pyke et al., 1994).

### Punctate protein localizations used as fiducial markers establish that any region of the plastid envelope might extend to form a stromule

**Figure 2:**
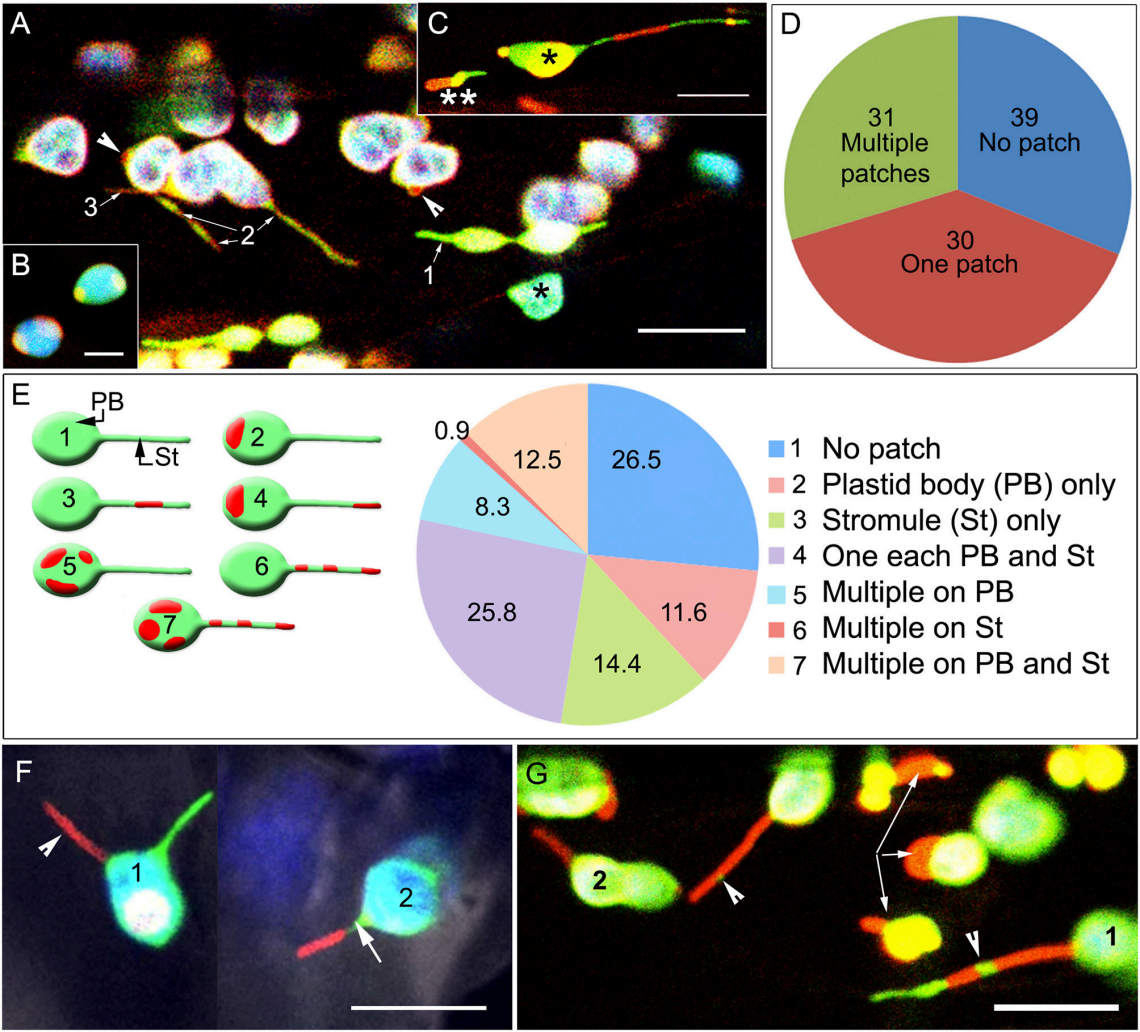
Spatial relationship between fluorescent fusion protein patches, the main plastid body (PB) and stromules (St)**. (A)** Representative image showing a transporter protein fusion in the tpFNR-EGFP background. Chloroplasts appear blue-green (_*_) due to chlorophyll auto-fluorescence (false-colored blue) and the stroma-targeted GFP (green). Transporter fusion protein patches are red fluorescent and may be absent (1), cover short lengths (2), or extend over an entire (3) stromule. **(B)** A representative image showing patch localization on the ends of lens-shaped mesophyll chloroplasts. **(C)** Image showing a chloroplast (^*^) and a leucoplast (^**^) in a hypocotyl cell in a seedling expressing the GPT1-mEosFP fusion (observed after photo-conversion). The chloroplast body (^*^) shows extensions on both sides. The varying mix of the green fluorescence of the stroma and the red fluorescence of the different sized transporter-fusion-protein patches results in their colors ranging from yellow to red. The leucoplast (^**^) exhibits an ectopic red bulge that differs from the green stromule extending on the opposite end of the plastid. In both plastids the higher intensity of red fluorescence suggests a region with low stroma filling. **(D)** Assessing 1624 chloroplasts of a TPT1-mEosFP line showed that 39.2% did not have a protein patch at all while nearly equal numbers of plastids had a single patch or several patches, 29.7 and 31.1%, respectively. **(E)** diagrammatic depiction, accompanying legend and pie diagram show the seven categories assessed for understanding the spatial relation between patches on the chloroplast body (PB) and extended stromules (St) in chloroplasts. Amongst plastids with extended stromules there could be no patches on the stromules (category 2) while one or more patches could be found on the PB (category 2, 5). Alternatively, one to several patches could be found on stromules (category 3, 6, 7) and none on the PB. Alternatively, one or more patches could be found randomly dispersed on both St and PB (category 7). **(F)** Two simultaneously extended stromules (1) in a chloroplast; one appearing red (arrowhead) due to extension of a region with a red fusion-protein patch and the other appearing green as it is devoid of any patch (Supplementary Movie 1). Chloroplast (2) shows a single stromule with the tip-region exhibiting an extended red patch while the proximal region (arrow) of the stromule devoid of a protein patch appears green. **(G)** The fusion-protein patches could localize randomly along the length of a stromule to form unlinked regions (e.g., 1) or appear as a single long region that highlighted an entire stromule (e.g., 2). The green fluorescent stroma, denoting a region of a stromule without a patch on the inner-envelope membrane appears as a small intervening region between the patches (arrowheads) or highlights a large portion of the extended stromule tip (e.g., 1). Arrows point to large misshapen patches that bulge outwards from the plastids. Size bars B, C = 5; A, F, G = 10 μm.

In each of the fiducial marker lines, the area covered by the protein punctae/patches varied from 1 to 12 μm^2^ with the larger red fluorescent patches frequently obscuring the underlying green-fluorescent stroma completely (Figure 2A). Pavement cell chloroplasts that are smaller than mesophyll chloroplasts and have a relatively low chlorophyll content (Barton et al., 2018), as well as leucoplasts, often appeared as orange-yellow due to the overlay of the green and red fluorescence from the FPs. In single snapshots a varying number of plastids in each cell exhibited ectopic bulges and misshapen tubular protrusions, where in some of these bulges/protrusions an internal green-fluorescent stroma was not detected or was present only at the far end of the protrusion (Figure 2B). The presence of patches resulted in a varying number of plastids in a cell showing a beaked appearance or displaying protein patches on opposite ends of the lens-shaped plastid body (Figure 2C). However, stromules were not always initiated from these fusion-protein enriched domains.

Of the 1,624 chloroplasts counted in the pro35S:TPT1-mEosFP 39.2% did not have a green to red fluorescent photo-convertible label, 29.7% had a single TPT1-mEosFP protein patch and 31.1% had several patches (Figure 2D). However, the number of chloroplasts extending stromules in soil grown plants after an end of night period averaged between 3 and 5% and was comparable to the parent tpFNR-GFP line. This suggested that over-expression of the TPT1 fusion protein did not affect the incidence of stromule formation.

In chloroplasts that extended stromules the punctae were located at different positions in relation to the extensions and were therefore placed into several broad categories (Figure 2E). These included no patch at all despite the presence of a stromule, one or more patches on the plastid body only, one or more patches on stromules only, and one or more patches on both plastid body and stromules (Figure 2E). Of the 1,543 chloroplasts exhibiting stromules, TPT1-mEosFP patches that localized to the plastid body or to an extended stromule were distributed nearly equally (Figure 2E). Whereas, sporadic protuberances and long extensions often formed from the undulating chloroplast envelope at any point, only a green fluorescent stromule was observed if the region of the envelope where it originated was free of a TPT1-mEosFP patch. Occasionally, two stromules were extended simultaneously from a chloroplast, and depending upon the position and the size of the TPT1-mEosFP patch one stromule could appear red while the other showed only the green stroma (Figure 2F; Supplementary Movie 1).

**Figure 3:**
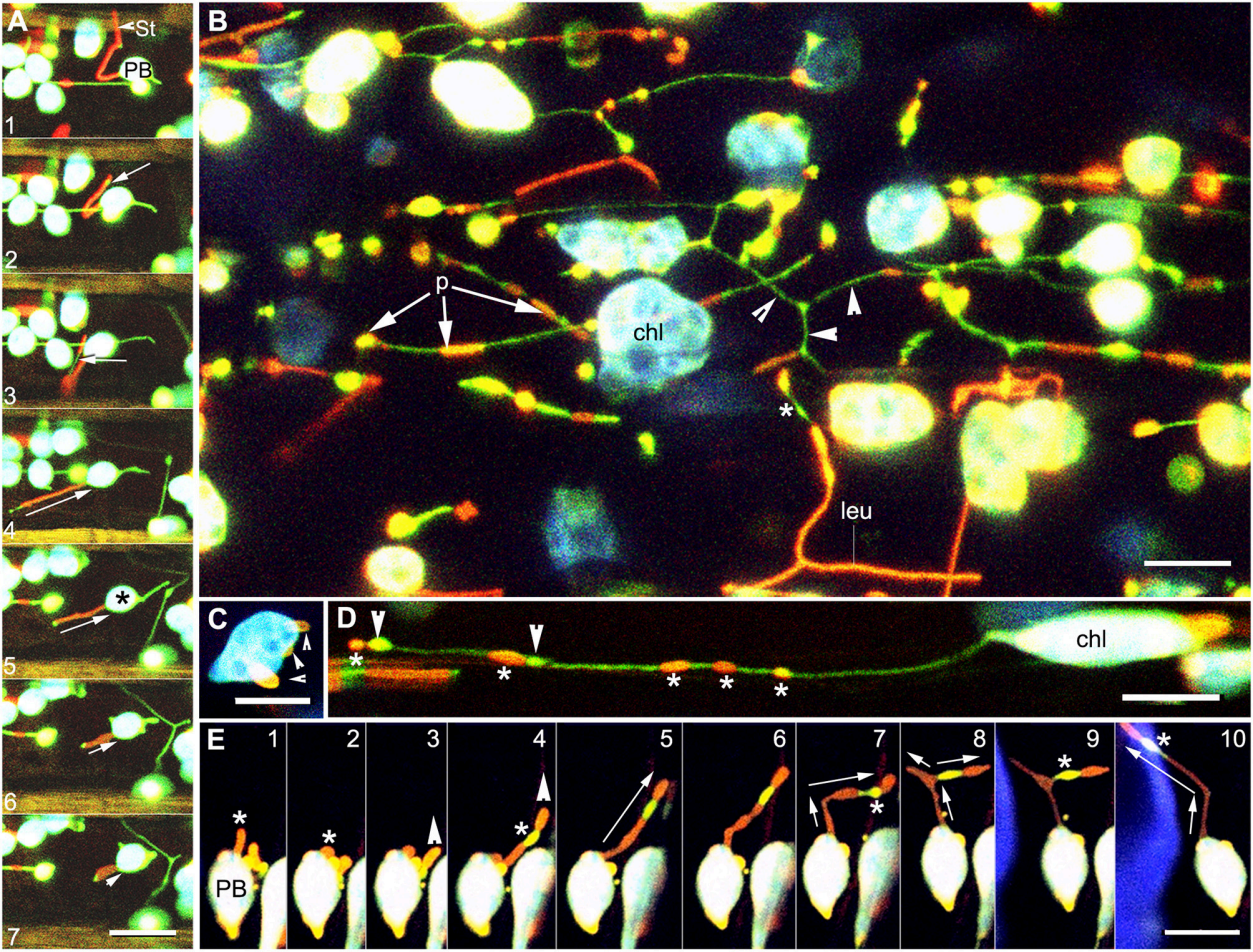
Images illustrating the changes in position and the diversity in extension of fluorescent protein patches on dynamic stromules. **(A)** Images from a time lapse sequence showing the change in patch size in a stromule retracting (relative sized directional arrows in frames 4–7) toward the plastid body (PB) establish that neither the position or the size of a protein patch on a stromule is fixed in relation to the PB. A small stromal region not covered by the red patch (arrow in frame 2) appears greatly elongated in frame 3 (arrow) to suggest two patches. Later frames 4–7 no longer show two patches but a single red fluorescent stromule with the tip devoid of a patch. Note a green stromule extended from the opposite side of the chloroplast (^*^) (Supplementary Movies 2, 3). **(B)** Representative image of the abnormally large chloroplasts (chl) and leucoplasts (leu) in hypocotyl cells of an *arc6* transgenic line expressing tpFNR-EGFP and GPT1-mEosFP. Following photo-conversion of mEosFP patches (p) of differing lengths and thickness are observed on both the plastid body and the stromules. Regions of a stromule without a patch appear green (arrowheads). **(C)** Image of a single *arc6* chloroplast showing three separate patches (arrowheads). **(D)** Stromule from a chloroplast (chl) showing GPT1-mEosFP patches (^*^) of variable size and fluorescence intensity. The stromule also shows two dilated regions (arrowheads) that are devoid of the mEosFP patch. **(E)** Ten images from a time-lapse sequence depict an extended tubule (^*^) in panel 1 and its retraction to the plastid body (PB) in panel 2. Panel 3 shows the extension of another region of the plastid and its elongation (panels 4–10), slight beading (panels 6, 7), and branching (arrows in panel 8, 9). Note the changing position of the intervening patch-free region (^*^) showing the green fluorescence of the underlying stroma (Supplementary Movie 4). Size bars = 10 μm.

Patches localized randomly along the length of a stromule as unlinked regions or as a single long region that could extend the entire length of a stromule (Figure 2G). Sequential time lapse imaging of extending and retracting stromules showed that the position of a protein patch on a stromule was not fixed and changed continuously in relation to the plastid body (Figure 3A; Supplementary Movie 2). Often a region of the envelope with a protein patch could extend and depending upon its length, one or more patches could appear to be localized to the stromule. However, upon retraction of the stromule the patch (s) could relocate to the plastid body.

While a high percentage of patches observed on stromules could have indicated a preferential localization of the TPT1 transporter fusion protein to the extensions, this was not observed. Similar observations were made on the GPT1-mEosFP transgenic line. Taken together our data strongly suggests that fusion-protein patches of different sizes might be found randomly scattered on the inner envelope-membrane and that their chances of becoming localized to a stromule are stochastic.

We speculated that the variability in size and number of patches as well as their stochastic localization to stromules would become even more apparent in large-sized chloroplasts and tested this in the *arc6* mutant.

### Protein patch size and numbers increases but their stochastic localization to stromules is maintained in the *arc6* mutant

The Arabidopsis *arc6* mutant has 1–3 large mesophyll chloroplasts that are nearly 20 times the size of chloroplasts in the wild type (Pyke et al., 1994) and exhibit multiple long stromules (Holzinger et al., 2008). The double transgenic line expressing tpFNR-EGFP and GPT1-mEosFP in the *arc6* background showed large mesophyll chloroplasts with numerous patches distributed randomly on the main body and the stromules (Figures 3B,C). The *arc6* double transgenic lines also exhibited abnormal sized epidermal chloroplasts and leucoplasts in pavement cells of leaves and hypocotyls. Very long stromules extending up to 50 μm from epidermal plastids often displayed a random array of green and red regions denoting the stroma and inner envelope-membrane localized GPT1-mEosFP, respectively (Figure 3B). The observation that different regions of the inner envelope-membrane with the protein patches showed differing intensities of red fluorescence suggested that the patch density varies in the different regions. When viewed in combination with the green fluorescence of the underlying stroma the denser patches and stromule regions with a low stromal content appeared bright red while thin patches usually appeared yellow-orange (Figure 3B). The random localization of the patches along the stromule length did not interfere with the often-observed localized dilation of stromules (Hanson and Sattarzadeh, 2011; Mathur et al., 2012). While we have not subjected the sporadic occurrence of the dilations to a statistical analysis, we did not find their preferential occurrence in regions of a stromule with the transporter fusion-protein patches. Indeed, as shown in (Figure 3D), the dilations appeared to be formed anywhere along the length of a stromule.

The idea that different regions of a plastid, with or without protein patches, can be drawn out was greatly reinforced by observing stromule formation from plastids with multiple patches (Figures 3D,E) in the *arc6* mutant. As shown in the sequential time lapse (Figure 3E) a red fluorescent patch gave rise to a red fluorescent stromule that could retract to the plastid body and be pulled out again to form a long tubule (Figure 3E; frame 1 vs. frames 4–10). The number of discrete transporter-protein patches on a specific stromule and their spatial relation to the plastid body could change considerably as the stromule extended further into the cytoplasm or retracted toward the plastid body (Figure 3E).

While the use of fluorescent punctae as discrete fiducial markers on the *arc6* plastid envelope reinforced the stochastic spatiotemporal relationship between a fusion protein patch and a stromule it still left open the possibility that conditions that increase the frequency of stromule formation in a cell might favor transporter-protein localization to the extensions. This was explored next.

### Stochastic protein localization is maintained following exogenous sucrose-induced increase in stromule formation

**Figure 4:**
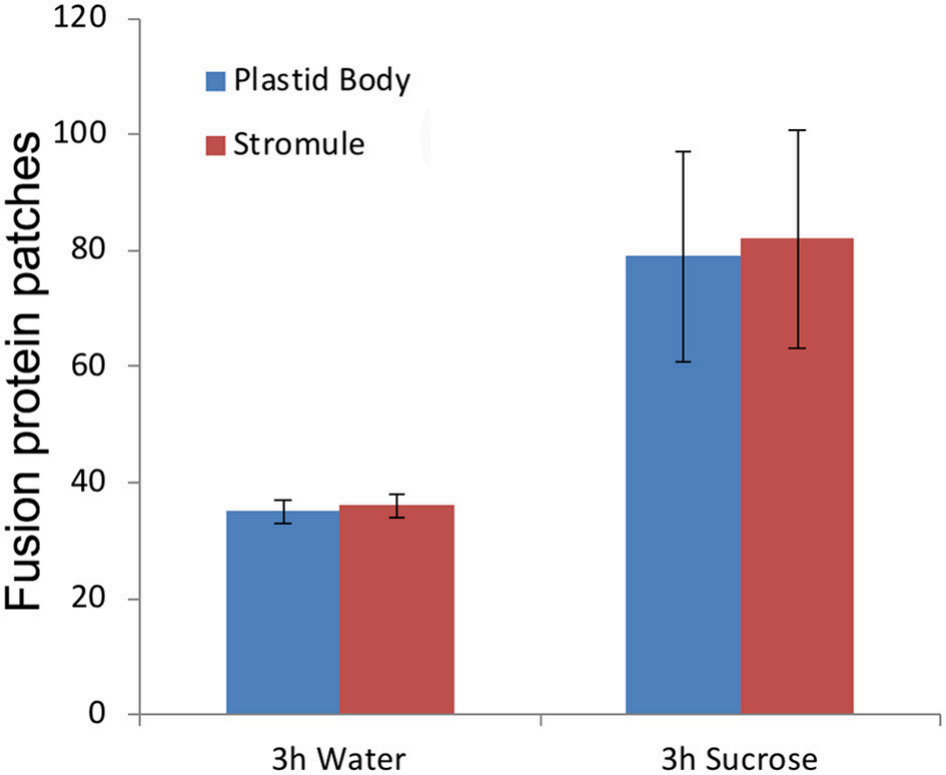
Exogenous feeding of seedlings with 40 mM sucrose results in more than 3-fold increase in percent stromule numbers per cell compared to the water controls. Despite increased stromule numbers the fluorescent TPT1-mEosFP fusion protein patches are evenly distributed between the plastid body and stromules. The representative data shown is from one experiment and is based on merged z-stack images from 16 cells (spread over 4 seedlings) per treatment (standard deviation shown). The data reinforces the chances of finding a patch anywhere on the plastid as stochastic.

Exogenous treatment of Arabidopsis seedlings with 40 mM sucrose in the dark leads to an increase in the number of plastids producing stromules (Schattat and Klösgen, 2011; Barton et al., 2018). Seven to ten-day old soil grown seedlings of pro35S:TPT1-mEosFP were taken after a 10-h dark period and checked for patches located on the plastid body and stromules. Most chloroplasts in the seedlings from the dark did not exhibit stromules but of the approximately 3% that did, a nearly equal number of patches were observed on the plastid body and the extended stromules. Subsequent treatment of the seedlings with 40 mM sucrose or water (control) for 3 h in the dark yielded a 25.3% stromule frequency in sucrose as compared to 7.6% in the controls. A statistical analysis of chloroplasts with extended stromules showed the protein patches evenly distributed between the plastid body and the stromules (Figure 4). A similar experiment carried out using a GPT1-mEosFP × tpFNR-EGFP double transgenic line and produced comparable results. We concluded that high stromule formation under sucrose treatment does not bias TPT1 or GPT1 localization specifically to stromules and maintains the stochastic relationship observed with lesser number of stromules per cell.

## Discussion

An increasing number of proteins are being reported as localizing to stromules. Notably, some of these proteins are being presented as stromule-specific with stromule-specific functions (Mueller and Reski, 2014; Mueller et al., 2014; Bross et al., 2017). While these reports are exciting they do not provide explanations for the suggested specificity of protein targeting to stromules. This study utilized plastid inner envelope-membrane localized proteins as fiducial markers to assess the possibility of a protein localizing to stromules and achieved the following insights.

### The use of different fluorescent protein fusions and the manner of protein expression can influence protein dispersal patterns on the plastid envelope

While we have not presented the results of transient overexpression of the different fluorescent protein fusions in detail, we became aware through our observations that this method can produce a mix of protein localization patterns that can then be interpreted in a very selective manner. The differences in protein dispersal patterns can be traced in some cases to the choice of fluorescent protein partner in the fusion protein. In the specific cases presented here we found that GPT1 fusions with EGFP resulted in a diffuse highlighting of the plastid envelope while fusion of GTP1 to mEosFP predominantly showed up as patches of varying sizes. Our observations also match earlier reports where similar patterns of protein dispersal have been described as GFP dots, and crescent moon-like, for inner envelope-membrane localized AtTIC40, a component of the TIC (Translocon of the Inner Chloroplast membrane) complex (Breuers et al., 2012). Similar to our observations the AtTIC40-GFP was found limited to small regions of the chloroplast inner envelope-membrane. An ultrastructural analysis of such patches shows local membrane proliferations of the inner envelope-membrane (Singh et al., 2008; Breuers et al., 2012) We concur with the suggestion by Breuers et al. (2012) made on the basis of an extensive analysis of transient expression of several plastid inner and outer envelope membrane proteins that the patches might be considered as artifacts of fusion protein over-expression.

It is known that the oligomerization of the many fusion proteins can also create a zippering effect that increases protein aggregation (Gong et al., 1996; Snapp et al., 2003). Notably, the monomeric version of EosFP used by us has been engineered from a naturally occurring tetrameric form Wiedenmann et al. (2004). While the mEosFP has been used successfully to label the plastid stroma, the mitochondrial matrix and cytoskeletal elements (Mathur et al., 2010; Schattat et al., 2012a,b), it is possible that its fusion to the membrane transporters and confinement to the inner envelope-membrane and / or the intermembrane space, leads somehow to artifactual protein aggregates. The tendency to form protein aggregates, especially in fusion with membrane proteins, has been reported for a different version of monomeric EosFP (Zhang et al., 2012). While not presented here, differences in protein dispersal patterns have also been observed by us upon screening transgenic lines expressing different fusion proteins with other monomeric fluorescent proteins such as monomeric red fluorescent protein (mRFP) created by Campbell et al. (2002).

Our observations underscore the importance of validating the punctate localization of a protein on plastids by checking more than one fluorescent protein partners in a fusion protein as well as considering the manner of protein expression. However, the fusion-protein artifact provided us with useful fiducial marker lines that allowed this investigation on protein localization to stromules to go further.

### Punctae used as fiducial markers for understanding protein localization in relation to stromules

As shown by our screening of different transporter lines the creation of stable transgenic lines does not automatically solve the artifact of patchy protein dispersal pattern. Similar observations of persistent fluorescent protein patches have been reported (Haswell and Meyerowitz, 2006; Aranda-Sicilia et al., 2016). Observations of single protein patches on chloroplasts highlighted by TPT1-mEosFP and GPT1-mEosFP often appeared as beaked plastids. We speculated that the protein patch representing the beak might serve as a point of stromule extension. However, this was found not to be the case. Further confirmation that stromule initiation is unlinked to the presence or absence of the protein patches came from observations of only green fluorescent stromules formed from chloroplasts with red colored protein patches in other locations. Further, analysis of the data for patch locations argued against the “stromule specific” nature of such patches. Patches could be found anywhere on the plastid envelope, including, but not limited to, the portion that was sporadically extended into a stromule. An equal distribution of patches on the extended stromules and the plastid body as well as observations of one or many randomly located patches on dynamic stromules reinforced the stochastic nature of the phenomenon.

Based on transient over-expression several proteins have been described as being stromule specific (Mueller et al., 2014; Bross et al., 2017). These include a *Physcomitrella* 3-ketoacyl-ACP reductase (KAR), a peroxiredoxin PpPrxQA (Mueller et al., 2014), five arogenate dehydratases (Bross et al., 2017), and a ferredoxin gene (Wang et al., 2017). Interestingly each of these proteins has been shown to target to the plastid stroma and therefore being found in stroma-filled tubules is neither surprising nor exclusive. The claim that these proteins are stromule-specific is also negated by the observations of Mueller et al. (2014), that KAR is present on both stromules and the main plastid body, while the PpPrxQA is distributed evenly between plastids and foci on stromules. Three other proteins that have been presented as stromule-specific are OEP16-2.2, OEP16-1.3, OEP16-2.1 (Mueller et al., 2014). As reported these are outer envelope-localized proteins whose GFP fusions localized only partly to long stromules but also to small protrusions on chloroplasts while showing low fluorescence in the plastid bodies (Mueller et al., 2014). Notably the over-expression of different outer envelope protein (OEP) fusions has been shown to result in ectopic tubular protrusions (Lee et al., 2001; Oikawa et al., 2008; Breuers et al., 2012; Machettira et al., 2012). As pointed out by Delfosse et al. (2016) protrusions made up of the outer envelope-membrane only do not constitute stromules, which by definition should contain both membranes of the envelope and a stroma filled tubule-interior (Köhler and Hanson, 2000). Based on the patterns reported by Mueller et al. (2014) and Bross et al. (2017) the localization of the proteins suggests a range rather than a specific localization.

The idea that there are stromule-specific proteins responsible for stromule-specific functions is mainly based on the idea that stromules constitute a plastid sub-compartment. This is discussed further.

### Stromules are sporadic and transient extensions and do not provide a post-translational protein targeting site

A vast majority of plastid proteins are post-translationally targeted to the organelle and become distributed between the plastid stroma, the thylakoid lumen and the different internal and envelope membranes (Jarvis, 2008; Sakamoto et al., 2008). In stark contrast to the relatively stable plastid components and compartments the occurrence of stromules is actually a sporadic and transient phenomenon (Natesan et al., 2005; Schattat et al., 2015; Hanson and Hines, 2018). Under normal conditions of plant growth and for a large portion of the diurnal cycle the majority of plastids in a cell do not exhibit stromules (Schattat and Klösgen, 2011; Brunkard et al., 2015). Even when formed from a plastid, these long tubules might remain extended for durations ranging from a few seconds to several minutes and subsequently retract to the plastid body. As such the transient extension and retraction of these thin tubules argues against their being exclusive for a sustained and essential plastidial function. However, extended stromules present a membrane topology, leaves open the possibility that proteins which prefer high-curvature environments might preferentially aggregate to these regions. Such proteins might serve to align stromules to cytoskeletal elements (Kwok and Hanson, 2004b; Erickson et al., 2018; Kumar et al., 2018), and provide membrane contact sites with other organelle membranes, including the endoplasmic reticulum (Schattat et al., 2011a,b).

The observations of selective concentration of “stromule proteins” leading to consequent “stromule-specific functions” (Mueller and Reski, 2014; Mueller et al., 2014) consider these tubules to be a plastid sub-compartment. While to the best of our knowledge a stromule-targeting amino acid sequence is not known, a search of the gene ontology data base AmiGO2 http://amigo.geneontology.org/amigo using the keyword stromule (GO:0010319) leads to 64 gene products with 58 of the proteins directly annotated as stromal. The Arabidopsis Information resource (TAIR; http://www.arabidopsis.org/) actually lists 39 records. Amongst these 33 protein records suggest stromule as the place of expression and are traceable to a single publication by Goulas et al. (2006). This key publication reports a proteomics approach to identify chloroplast lumen and stromal proteins during response to low temperatures and acclimation but does not mention the term stromule at all (Goulas et al., 2006).

At this stage, it is pertinent to ask whether the stromule, a stroma-filled tubule, constitutes a true, sustained plastidial sub-compartment. It is also relevant to consider where a protein that is considered as stromule-specific might be found in the majority of plastids in the absence of the transient stromule. That specific sub-organellar location, and not the transient stromule, would constitute the correct area of protein localization. Moreover, if we must persist in ascribing a function to stromules, we should first consider whether a protein of interest is capable of fulfilling its role while located on the plastid body as well and rule out such a possibility before declaring it as stromule specific. It is possible that we may in the future uncover a protein which is more active on the highly curved surface of a stromule rather than the relatively more planar plastid body. However, given the continuity of these membrane surfaces this appears unlikely. At most, for some specific protein one might be able to conclude that its presence on a stromule modulates its activity.

## Conclusions

We tested the idea of stromule specific localization for proteins by using fluorescent fusion protein patches on chloroplasts as fiducial markers to show the relationship between the main plastid body and its extensions. Based on observations of GPT1 and TPT1, two sugar-phosphate transporters, in a few stable transgenic lines, we have concluded that the discrete patches are not restricted to any specific location on a plastid. There is an equal likelihood of a patch being localized on the chloroplast body or a stromule. Our data strongly suggests that the localization of a protein to a stromule might reflect a stochastic phenomenon, and therefore, should not automatically be considered indicative of a stromule-specific function. The fusion protein localization data presented here is limited to a particular subset of inner envelope-membrane localized proteins and do not negate the possibility that some other, as yet unknown proteins or protein complexes, might localize to discrete regions on the envelope-membranes to create sites favorable for stromule initiation. Identifying such proteins and establishing its clear spatiotemporal relationship with the site of stromule initiation will certainly pave the way for a mechanistic understanding of the phenomenon. Based on our present observations we consider stromules as extensions that fulfill the same functions as the rest of the plastid's envelope and stroma yet allow these functions to be extended into a larger volume of the cytoplasm than can be reached by a single plastid without a stromule.

## Materials and methods

### Gene cloning

The full-length coding sequences for the Arabidopsis GPT1 (At5g54800) TPT1 (At5g46110), were obtained by PCR from a cDNA library and used to create C-terminal fusions with Green Fluorescent Protein (EGFP; Clontech; 6082-1), and monomeric EosFP (Wiedenmann et al., 2004), in a pCAMBIA 1300 vector background (http://www.cambia.org/daisy/cambia/585.html). The chimeric vectors were introduced in *Agrobacterium tumefaciens* GV3101 through electroporation.

### Plant material

All transient *Agrobacterium* infiltration-based experiments were carried out in leaves from 4 to 6 weeks day old plants of *Nicotiana benthamiana* (NB). Experiments aimed at understanding fusion-protein localizations in relation to stromules used stable transgenic lines *Arabidopsis thaliana* (Columbia ecotype) expressing the plastid stroma-targeted tpFNR-EGFP (Marques et al., 2003) as the background. All stable Arabidopsis transgenic plants were created by the *Agrobacterium* floral dip method (Clough and Bent, 1998) and selecting on 50 μM Hygromycin (H385; PhytoTechnology Labs; https://phytotechlab.com/).

The *arc6* mutant (Pyke et al., 1994) was transformed with tpFNR-EGFP (Marques et al., 2003) and a stable F3 transgenic line transformed with pro35S:GPT1-mEosFP to obtain double transgenic *arc6* plants expressing stroma targeted tpFNR-EGFP and a photo-convertible GPT-mEosFP. Double transgenic lines expressing both proGPT1:GPT1-EGFP and the pro35S:TPT1-mEosFP were obtained by crossing and stabilized over three generations.

Arabidopsis seeds were germinated on Murashige and Skoog's medium (Murashige and Skoog, 1962) containing Gamborg B5 vitamins (M404; PhytoTechnology labs) and 3 g/L of Phytagel (Sigma-Aldrich), supplemented with 3% sucrose and with the pH adjusted to 5.8 before autoclaving. All seeds were stratified for 2 days at 4°C.

### Exogenous treatments with sucrose

A treatment with 40 mM sucrose solution has been used to increase the stromule induction frequency from plastids (Schattat and Klösgen, 2011). However, instead of excised leaf-discs we dipped entire 7–10 day old seedlings, grown in soil under 100 μmol m^−2^ s^−1^ white light and 21°C ambient temperature. Seedlings were placed in the sucrose solution and incubated in the dark for 3 h before being viewed in the same solution on glass depression slides.

### Microscopy and data processing

A three-channel Leica TCS-SP5 confocal laser-scanning unit equipped with 488 nm Ar and 543 nm He-Ne lasers was used. The green to red photo-convertible mEosFP was irreversibly converted to exhibit red fluorescence following an exposure of 5 s with violet blue light (Leica D-filter; Ex, BP 355–425; dichroic 455; LP 470 nm; Mathur et al., 2010). Emission spectra acquired were: EGFP−503 to 515 nm (green); mEosFP- 585 to 630 nm (red); Chlorophyll−650 to 710 nm (false colored blue). Since photo-conversion of mEosFP was limited to 5 s an overlap between the green and red spectra often resulted in a yellow-orange color.

All images were captured at a color depth of 24 bit RGB. All images and movies were annotated, cropped and processed for brightness / contrast as complete montages or image stacks using either Adobe Photoshop CS3 (http://www.adobe.com) or the ImageJ / Fiji platform (https://fiji.sc/).

### Statistical analysis

Stromule counts were performed on image z-stacks taken on the hypocotyls of 7-day-old *A. thaliana* seedlings. Four images were taken for each of four seedlings per treatment. As described by Köhler and Hanson (2000), a stromule was considered as a thin tubule extending away from the plastid body. Ectopic protrusions where the tpFNR-GFP-highlighted stroma was not visible were not considered in our stromule counts. Two-tailed *t*-tests were made to determine the significance of results. Significance was pre-determined as having a *p* < 0.01 (99% confidence interval).

## Author contributions

JM conceived and supervised the research. KD, MW, KB, and NG created the different gene fusions and helped with imaging and data collection. NM provided technical assistance. KD and KB analyzed the data. All authors contributed to the writing of the manuscript.

### Conflict of interest statement

The authors declare that the research was conducted in the absence of any commercial or financial relationships that could be construed as a potential conflict of interest.

## Supplementary material

Supplementary Movie 1 Time lapse sequence of two chloroplasts used as basis for Figure 2F shows that stromules as well as portions of stromules might appear different due to the presence of protein patches in the region of the plastid envelope that extended.

Supplementary Movie 2 Time-lapse sequence showing relative positional changes that may occur as different regions of the plastid envelope with (red; GPT1-mEosFP) and without (green stroma only) a fluorescent protein patch are extended, retracted and rotated as part of chloroplast movement during cytoplasmic streaming.

Supplementary Movie 3 Time-lapse sequence used as basis for Figure 3A reinforces the continually changing spatiotemporal relationship between the dynamic, extending and retracting stromules and the main plastid body.

Supplementary Movie 4 Time-lapse sequence used as basis for Figure 3E depicts the inconsistency of shape and position of a patch on a stromule in relation to the main plastid body.
